# Super-resolution sodium MRI of human gliomas at 3T using physics-based generative artificial intelligence

**DOI:** 10.1007/s11060-025-05094-x

**Published:** 2025-06-03

**Authors:** Catalina Raymond, Jingwen Yao, Alfredo L. Lopez Kolkovsky, Thorsten Feiweier, Bryan Clifford, Heiko Meyer, Xiaodong Zhong, Fei Han, Nicholas S. Cho, Francesco Sanvito, Sonoko Oshima, Noriko Salamon, Linda M. Liau, Kunal S. Patel, Richard G. Everson, Timothy F. Cloughesy, Benjamin M. Ellingson

**Affiliations:** 1 UCLA Brain Tumor Imaging Laboratory (BTIL), Departments of Radiological Sciences, Psychiatry, and Neurosurgery, David Geffen School of Medicine, Center for Computer Vision and Imaging Biomarkers, University of California, Los Angeles, 924 Westwood Blvd., Suite 615, Los Angeles, CA 90024 USA; 2 Department of Radiological Sciences, David Geffen School of Medicine, University of California Los Angeles, Los Angeles, CA USA; 3 CEA, NeuroSpin, CNRS, Paris-Saclay University, Gif-sur-Yvette, France; 4 Research and Clinical Translation, Magnetic Resonance, Siemens Healthineers AG, Erlangen, Germany; 5 Siemens Medical Solutions USA, Inc., Boston, MA USA; 6 Department of Bioengineering, Henry Samueli School of Engineering and Applied Science, University of California, Los Angeles, Los Angeles, CA USA; 7 Medical Scientist Training Program, David Geffen School of Medicine, University of California, Los Angeles, Los Angeles, CA USA; 8 Department of Neurosurgery, David Geffen School of Medicine, University of California Los Angeles, Los Angeles, CA USA; 9 Department of Neurology, David Geffen School of Medicine, University of California Los Angeles, Los Angeles, CA USA

**Keywords:** Sodium MRI, Brain tumors, Generative adversarial network, Glioma, Image reconstruction

## Abstract

**Purpose:**

Sodium neuroimaging provides unique insights into the cellular and metabolic properties of brain tumors. However, at 3T, sodium neuroimaging MRI’s low signal-to-noise ratio (SNR) and resolution discourages routine clinical use. We evaluated the recently developed *A*natomically cons*t*rained GAN using p*h*ysics-bas*e*d sy*n*thetic MRI *a*rtifacts” (ATHENA) for high-resolution sodium neuroimaging of brain tumors at 3T. We hypothesized the model would improve the image quality while preserving the inherent sodium information.

**Methods:**

4,573 proton MRI scans from 1,390 suspected brain tumor patients were used for training. Sodium and proton MRI datasets from Twenty glioma patients were collected for validation. Twenty-four image-guided biopsies from seven patients were available for sodium-proton exchanger (NHE1) expression evaluation on immunohistochemistry. High-resolution synthetic sodium images were generated using the ATHENA model, then compared to native sodium MRI and NHE1 protein expression from image-guided biopsy samples.

**Results:**

The ATHENA produced synthetic-sodium MR with significantly improved SNR (native SNR 18.20 ± 7.04; synthetic SNR 23.83 ± 9.33, *P* = 0.0079). The synthetic-sodium values were consistent with the native measurements (*P = 0.2058*), with a strong linear correlation within contrast-enhancing areas of the tumor (*R*^*2*^ *= 0.7565*,* P = 0.0005*), T2-hyperintense (*R*^*2*^ *= 0.7325*,* P < 0.0001*), and necrotic areas (*R*^*2*^ *= 0.7678*,* P < 0.0001*). The synthetic-sodium MR and the relative NHE1 expression from image-guided biopsies were better correlated for the synthetic (*ρ* *= 0.3269*,* P < 0.0001*) than the native (*ρ* *= 0.1732*,* P = 0.0276*) with higher sodium signal in samples expressing elevated NHE1 (*P < 0.0001*).

**Conclusion:**

ATHENA generates high-resolution synthetic-sodium MRI at 3T, enabling clinically attainable multinuclear imaging for brain tumors that retain the inherent information from the native sodium. The resulting synthetic sodium significantly correlates with tissue expression, potentially supporting its utility as a non-invasive marker of underlying sodium homeostasis in brain tumors.

**Supplementary Information:**

The online version contains supplementary material available at 10.1007/s11060-025-05094-x.

## Introduction

Sodium (²³Na) neuroimaging is a promising technique for the non-invasive assessment of brain tumor physiology, with applications in both diagnosis and monitoring. Unlike traditional proton MRI, which primarily reflects water-based tissue contrasts, sodium MRI provides unique insights into the cellular microenvironment and metabolic status of tissues [1–5]. Brain tumors, particularly aggressive forms like glioblastomas, often exhibit altered sodium concentrations due to increased cellularity, disrupted membrane ion transport, and elevated metabolic activity [6, 7]. This makes sodium MRI a valuable tool for capturing the biochemical landscape of tumors, potentially aiding in the evaluation of tumor aggressiveness and response to therapies.

In brain tumors, changes in sodium concentration are linked to key cellular processes such as cell proliferation, altered ion transport, and disrupted metabolism. Studies have shown that malignant gliomas exhibit elevated intracellular sodium level, thought to result from the upregulation of the sodium-proton exchanger (NHE1) and sodium-potassium pumps due to increased metabolic demand [2, 8–10]. Malignant gliomas may also exhibit increased extracellular fraction related to extracellular matrix reorganization and increased vascular permeability, contributing to a total increase in tissue sodium concentration. Such sodium shifts can provide indirect markers of tumor cellularity, edema, and necrosis, which are critical in assessing tumor progression and treatment response [11, 12].

Despite its clinical potential, sodium neuroimaging at 3T faces substantial limitations, primarily due to its intrinsically low sensitivity and fast T_2_ decay in tissue, limiting the spatial imaging resolution that can be achieved and hindering its adoption for routine clinical use. Sodium ions have a lower MR sensitivity than protons, present a quadrupolar moment leading to a fast T_2_ relaxation of the sodium signal in biological tissue [13] and the low concentration of sodium in the body results in a low signal-to-noise ratio (SNR), making it challenging to capture high-resolution images without extended scan times. Consequently, sodium MRI performed at 3T often suffers from lower spatial fidelity and partial volume effects, complicating its interpretation, tissue sodium quantification and reducing its clinical reliability [6, 7].

Previous efforts to improve sodium imaging have focused primarily on hardware-based solutions, such as leveraging ultrahigh magnetic field strength [7, 14], utilizing, or implementing ultra-short echo time (TE) acquisitions with non-Cartesian k-space sampling schemes [15–17]. In clinical 3T systems, sodium imaging also requires dedicated hardware support, including custom radiofrequency coil designs [18, 19], an additional radiofrequency power amplifier, and the multinuclear imaging option, all of which contribute to limited accessibility and clinical adoption. While these approaches can improve image quality, they often come with increased cost, technical complexity, and limited accessibility in standard clinical environments as demonstrated by Nagel et al. [7] who reported improved sodium imaging at 7T but acknowledged the limited availability of such systems for routine clinical use.

Advanced computational methods, including deep learning, have been increasingly applied to enhance MRI resolution and reduce artifacts. Generative adversarial networks (GANs) have demonstrated potential in MRI applications by enabling high-resolution image reconstructions, even from low-SNR data, with promising results in various domains such as cardiac, musculoskeletal, and neuroimaging [20–23]. Physics-informed GANs incorporate known imaging physics into the pre-processing and model to improve reconstruction accuracy while preserving anatomical integrity [24–27]. However, while GAN-based approaches have been applied in proton MRI artifact correction and resolution enhancement, their use in sodium MRI, specifically for brain tumor imaging is limited [28].

This study employs an **A**natomically cons**t**rained GAN using p**h**ysics-bas**e**d sy**n**thetic MRI **a**rtifacts” (ATHENA) model [29] to enhance sodium MRI quality at 3T, enabling high-resolution synthetic-sodium MR images for brain tumor imaging. We adopted the recently developed ATHENA model [29], which was based on a modified Pix2Pix GAN architecture with stacked transfer learning and has demonstrated efficacy in recovering anatomical detail and mitigating artifacts in proton echo-planar imaging (EPI). The ATHENA leveraged a highly diverse training data comprising multiple proton MRI contrasts to improve its generalization across a range of imaging scenarios. Additionally, the incorporation of anatomical constraints improved the model’s ability to correct artifacts while preserving key anatomical and tissue-specific information. The model has been validated on a dataset of proton apparent diffusion coefficient (ADC) maps, a contrast absent in the training data, highlighting its generalizability [29]. We hypothesize that the ATHENA model will enable the robust synthesis of high-resolution high-SNR sodium images while maintaining the underlying biological information essential for clinical interpretation, as validated through correlations with native sodium MR. The robustness and clinical relevance of this approach are further demonstrated by comparing the high-resolution synthetic data with the immunohistochemical expression of the NHE1 in glioma tissue samples.

## Materials and methods

To evaluate the performance of the ATHENA model in the context of sodium MRI, synthetic high-resolution sodium MR images were generated for a validation cohort of 20 patients. During inference, the model took as input the native low-resolution sodium MR images along with edge information extracted from post-contrast T1-weighted anatomical scans using the Canny edge detection algorithm. While originally developed for correcting artifacts in echo-planar imaging (EPI) [29], in this study, ATHENA was repurposed here to enhance sodium MRI with the goal of improving spatial resolution and signal-to-noise ratio (SNR). Importantly, the model was not trained on any native sodium data. Instead, ATHENA was trained entirely on artifact-free proton MRI scans with synthetic artifact augmentation, relying on the structural priors learned from anatomical images. Despite this, we hypothesized that the model would generalize to sodium data and produce biologically faithful reconstructions—a hypothesis supported by the strong correlation between the synthesized images and the original sodium MR scans.

### Synthetic data generation and artifact reduction

To train ATHENA, synthetic MRI datasets were created by simulating common clinical MRI artifacts using physics-based simulations from 4,573 artifact-free MRI scans acquired in 1,390 brain tumor patients who underwent standard 3T proton MRI examinations [30]. MRI scans included high-resolution pre- and post-contrast T1-weighted, T2-weighted, and T2-weighted fluid-attenuated inversion recovery (FLAIR) images acquired following established clinical guidelines (see Supplemental Table 1 for acquisition details). Simulated artifacts included: (1) modeling *B*_*0*_ magnetic susceptibility-related geometric artifacts [31, 32], (2) chemical shift artifacts based on fat-water frequency differences at 3T (430 Hz) [33], (3) Nyquist aliasing artifacts, (4) Gibbs ringing and image downsampling, (5) and various levels of Rician noise. Details on these various artifacts and implementations can be found in Raymond et al. [29].

### GAN model development and training

**Fig. 1:**
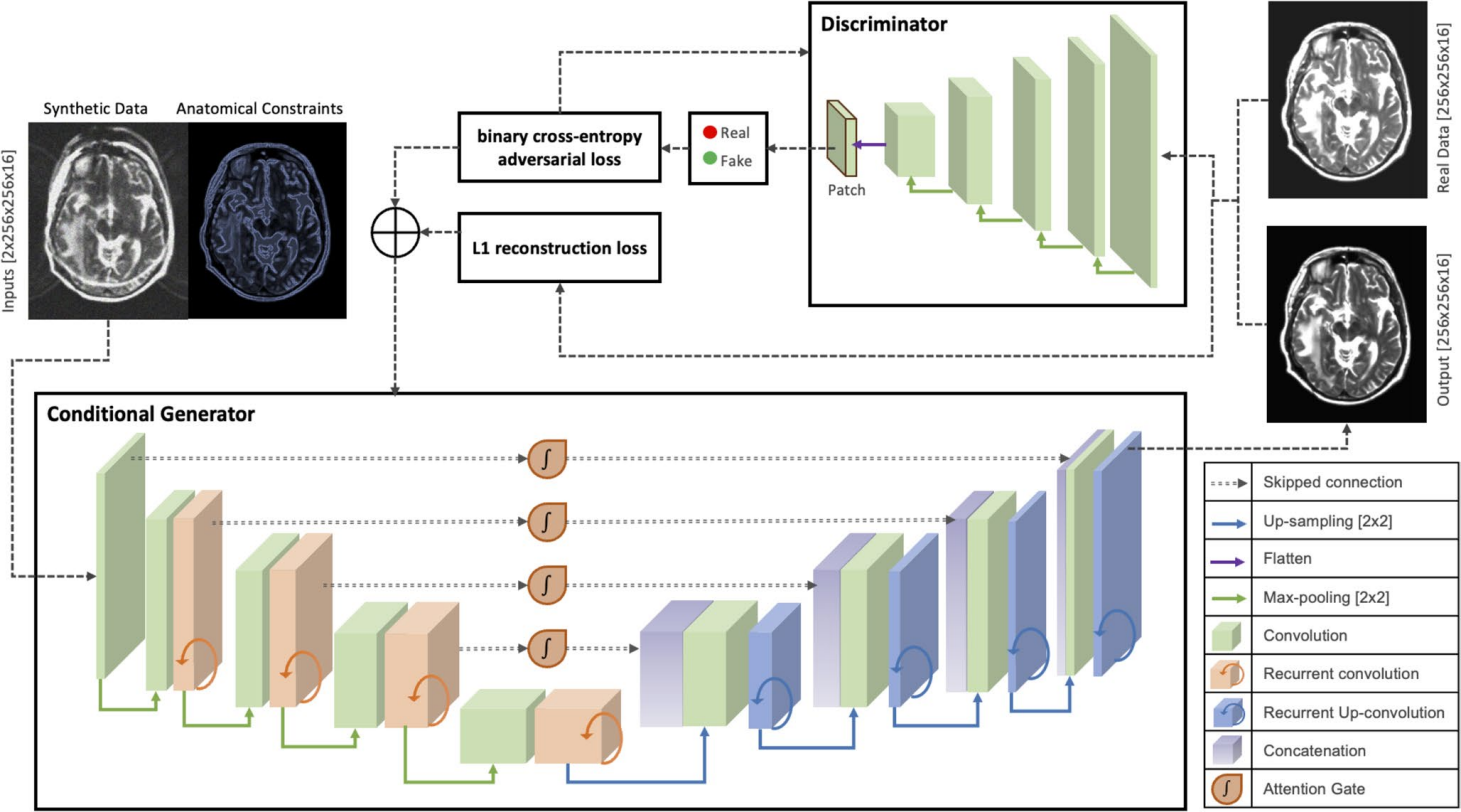
Generative adversarial network (GAN) schematic: attention-R2UNet generator network architecture and PatchGan discriminator architecture

The ATHENA model (Fig. 1) consisted of a physics-informed generative adversarial network (GAN) using a modified Pix2Pix architecture [34] with an Attention R2UNet generator [35–37]. Training utilized a stacked transfer learning approach, with models trained sequentially on increasingly complex artifacts (Supplemental Fig. 1). The network was trained with paired synthetic and artifact-free images, integrating edge-detected images using the Canny algorithm [38] to maintain anatomic accuracy. The model used a 3D PatchGAN discriminator architecture with each convolutional layer using a 3 × 3 × 3 kernel with stride of 2 and padding of 1. This configuration, spanning 4 layers, yielded a receptive field of 31 × 31 × 16. The initial learning rate was set to 1 × 10^− 4^ and the Adam optimizer [39] was used to optimize model weights. The loss function combined binary cross-entropy and L1 reconstruction loss, using a weighting ratio of 1:200, and training was conducted over 50 epochs. Implementation of ATHENA was done in *PyTorch* on NVIDIA Tesla V100 GPUs, processing 256 × 256 × 128 volumes in 16-slice batches.

### Clinical ^1^H and ^23^Na datasets

Twenty (55% male and 45% female) glioma patients (55% GBMs) classified according to the 2021 World Health Organization (WHO) classification of central nervous system tumors [40] were used in this study. The study population was primarily composed of individuals newly diagnosed (55%). All patients provided written informed consent, and the study was approved by our institutional IRB as part of a prospective surgical study (IRB 21–000514). Table 1 presents the demographic characteristics of the sodium dataset.

**Table 1:** Demographic characteristics of in-vivo sodium validation set

Case	Age	Sex	Diagnosis	Tumor Grade	Recurrence	Hemisphere	Lobe	MGMT	IDH Status	1p/19q Codeletion	EGFR Amplification	CDKN2A Loss	# Biopsies
1	52	M	Glioblastoma	4	New	Left	Temporal	promoter methylated	WT	N/A	+	Loss	4
2	46	M	Oligodendroglioma	2	Recurrent	Bilateral	Frontal	unmethylated	MUT	+	N/A	Intact	N/A
3	65	F	Glioblastoma	4	New	Right	Intraventricular	unmethylated	WT	N/A	+	N/A	N/A
4	60	M	Glioblastoma	4	Recurrent	Right	Temporal	unmethylated	WT	N/A	+	N/A	2
5	46	F	Glioblastoma	4	New	Right	Temporal	unmethylated	WT	N/A	-	N/A	N/A
6	27	F	Oligodendroglioma	2	New	Right	Frontal	unmethylated	MUT	+	N/A	Intact	N/A
7	39	M	Astrocytoma	4	Recurrent	Left	Temporal	unmethylated	MUT	-	-	N/A	N/A
8	44	M	Glioblastoma	4	Recurrent	Left	Occipital	unmethylated	WT	-	-	Loss	4
9	37	M	Glioblastoma	4	Recurrent	Left	Parietal	unmethylated	WT	N/A	+	Loss	N/A
10	60	M	Glioblastoma	4	Recurrent	right	parieto-occipital	unmethylated	WT	-	+	Loss	3
11	35	F	Glioblastoma	4	New	Left	frontoparietal	promoter methylated	WT	-	+	Intact	3
12	40	M	Astrocytoma	2	Recurrent	Right	Parietal	unmethylated	MUT	-	N/A	Intact	3
13	29	F	Astrocytoma	2	New	Left	parieto-occipital	promoter methylated	MUT	N/A	N/A	Intact	N/A
14	35	F	Astrocytoma	2	New	Right	Parietal	promoter methylated	MUT	-	N/A	Intact	5
15	50	F	Glioblastoma	4	New	Left	Thalamus	unmethylated	WT	N/A	-	N/A	N/A
16	37	F	Anaplastic Oligodendroglioma	3	Recurrent	Left	Parietal	promoter methylated	MUT	+	N/A	Intact	N/A
17	55	M	Glioblastoma	4	New	Left	Temporal	unmethylated	WT	-	-	Loss	N/A
18	46	M	Astrocytoma	2	New	Right	Frontal	promoter methylated	MUT	-	N/A	Intact	N/A
19	78	F	Glioblastoma	4	New	Right	Temporal	promoter methylated	WT	+	+	N/A	N/A
20	58	M	Glioblastoma	4	Recurrent	Right	Parietal	promoter methylated	WT	N/A	+	Intact	N/A

All patients were scanned on a 3T MRI system (MAGNETOM PrismaFit, Siemens Healthineers AG, Forchheim, Erlangen, Germany) prior to surgical resection between October 2021 and October 2023. Proton and sodium acquisitions were conducted during the same session using a dual-tuned head volume coil (16-channel ^1^H/1-channel ^23^Na; RAPID MR International; Columbus, OH). Anatomical pre-/post-contrast high-resolution T1-weighted, T2-weighted, T2/FLAIR, and diffusion-weighted (DWI) images were obtained following the guidelines of the standard brain tumor imaging protocol [30]. Sodium MRI was performed using a 3D spoiled gradient-echo sequence optimized for short TE with parameters: TE/TR = 2.39/10.52 ms, 5.5 mm isotropic resolution, 264 × 264 × 264 mm^3^ FOV, ~ 39.8^o^ flip angle (global B1 RF power transmit calibration with increasing flip angles each subject), 80 Hz/pixel bandwidth, 26 averages, and 10.5 min total scan time.

### Imaging-guided stereotactic biopsy and immunohistochemistry

Image-guided biopsies were obtained from eight patients in the study cohort to evaluate the expression of the sodium-proton exchanger (NHE1) through immunohistochemistry. We obtained 8–10 mm diameter spherical stereotactic image-guided biopsy samples of tumor tissue in regions with distinct metabolic characteristics in these patients during their clinically indicated surgical resection. Immediately following the biopsy, the tissue underwent quantitative IHC: samples were flash-frozen, sliced, placed onto slides, and then labeled and stained for NHE1 (S-A, HPA052891) expression. Tissue slides were scanned using a digital slide scanner (Aperio CS2, Leica Biosystems Imaging, Inc, Buffalo Grove, IL United States). The percentage of positive cells was calculated as the ratio of the number of positive cells to total cells in a tissue section, using a semi-automated positive cell detection algorithm implemented in QuPath ver. 0.2.0-m8 [41]. The degree of NHE1 expression was ranked from 0 to 3, where 0 - no expression, 1 (mild)– up to 33% expression, 2 (moderate)– between 33 and 66% expression, and 3 (strong)– between 66% and 100% expression based on quantitation and visual inspection by investigators. “Elevated” NHE1 expression was considered any biopsy showing NHE1 expression (or scoring 1–3).

### Image analysis

High-resolution (1 × 1 × 1 mm) synthetic-sodium MR images were generated using ATHENA for all 20 patients in the validation cohort, using the original sodium MR images and edge information extracted from post-contrast T1-weighted images via the Canny algorithm as inputs to ATHENA. Two tumor volumes of interest (VOIs) were segmented: contrast-enhancing tumor (CET), necrosis and T2 hyperintense regions. Segmentation of tumor regions was performed semi-automatically with guidance from NS-HGlio (Neosoma Inc, Groton, MA, https://neosomainc.com) [42] and then edited by a board-certified neuroradiologist to exclude any nontumor tissue. Supplemental Fig. 2 shows an example case.

To assess the accuracy of the synthetic images, native and synthetic-sodium images were normalized to the mean intensity of a VOI in the vitreous humor as described in a prior study [43]. The VOIs were manually placed as 5 mm diameter spheres in the center of both the left and right eyes. Finally, the normalized mean signal of the contrast-enhancing tumor, necrosis and T2 hyperintense regions was calculated for statistical analysis.

The ^23^Na SNR was calculated from the sodium images by comparing the mean signal intensity within the T2 hyperintense region to the standard deviation of signal intensity within a normal-appearing white matter (NAWM) mask. The NAWM mask was selected to represent a region with consistent signal intensity and no visible abnormalities, serving as a proxy for background noise. Additionally, SNR was independently calculated within NAWM using the ratio of its mean signal intensity to its standard deviation. This dual approach ensures that the calculated SNR captures both the contrast between pathological and normal tissue as well as overall image quality within healthy brain regions, providing a robust metric for assessing diagnostic utility.

### Statistical analysis

Correlation analyses were performed between the synthetic and native sodium MR measurements within the T2 hyperintensity, necrotic and contrast-enhancing tumor regions using Pearson’s correlation. Paired comparisons between the tumor ROIs synthetic and native sodium values were conducted using paired t-tests. Similarly, paired t-tests were used to compare SNR between the synthetic and native sodium values within T2 hyperintensity and NAWM ROIs. The sodium MR signal intensities were compared between tumor regions with high and low NHE1 expression levels using Student t-test, and correlation analyses with NHE1 expression level was performed using Spearman’s correlation for both native and synthetic-sodium. All values were reported as mean ± standard deviation (SD). A *p*-value of < 0.05 was considered significant.

## Results

A total of 20 patients (Table 1) were included in the study, with a mean age of 46.95 years (SD ± 12.76) and an age range of 27 to 78 years. The cohort comprised 11 males (55%) and nine females (45%). Among the patients, 11 (55%) were diagnosed with glioblastoma, while 9 (45%) had low-grade gliomas. Additionally, 11 patients (55%) were newly diagnosed, whereas 9 (45%) had recurrent tumors. Tumor localization varied across the cohort, with nine patients (45%) having tumors in the left hemisphere, eight patients (40%) in the right hemisphere, and one patient (5%) presenting with bilateral involvement.

### Image quality improvement

**Fig. 2:**
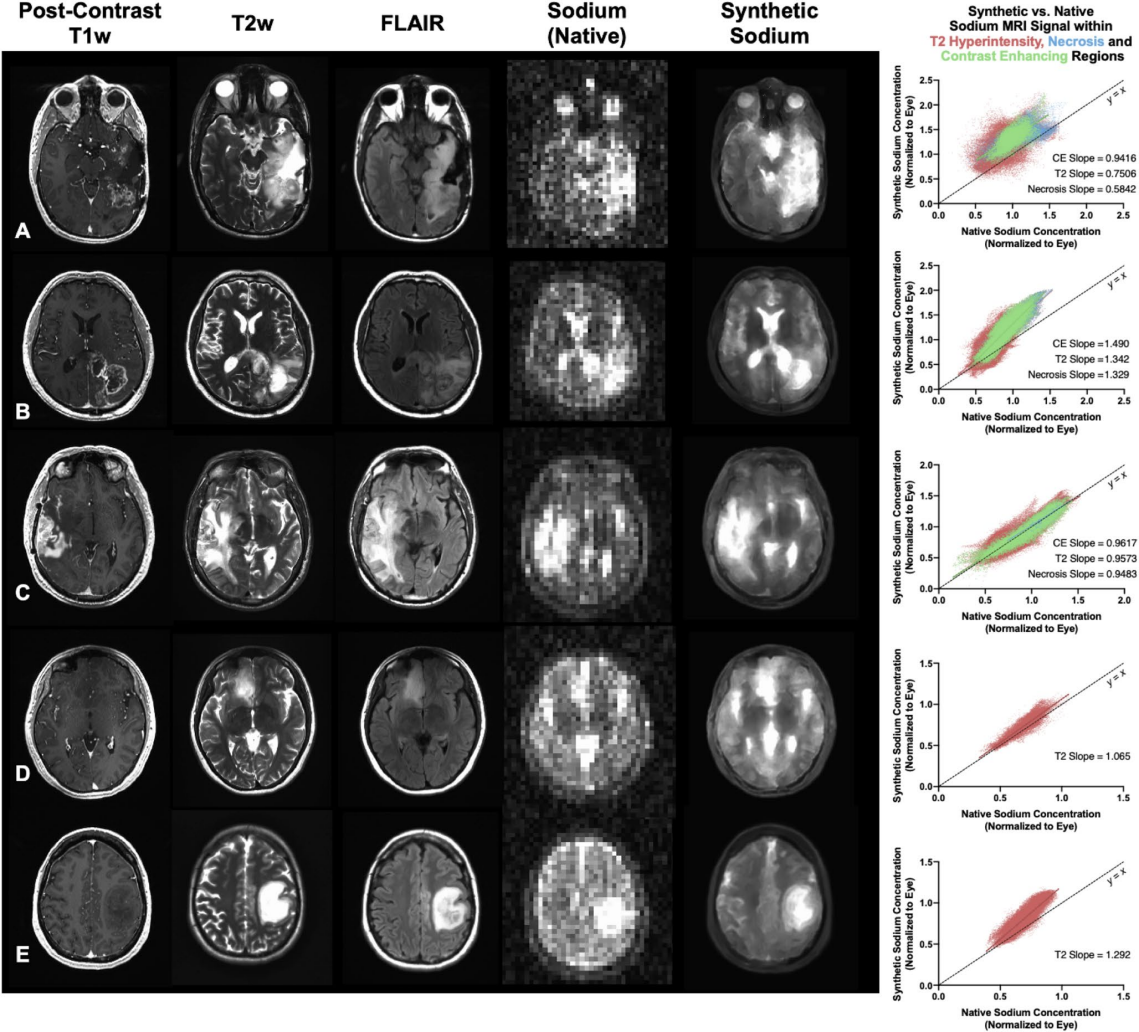
(**Left panel**) Post-contrast T1-weighted, T2-weighted, T2-weighted FLAIR, native 3T sodium MR images, high-resolution synthetic-sodium MR images and (**Right column**) voxel-wise correlation between native and synthetic sodium MR images for (**A-C**) patients with recurrent glioblastoma and (**D-E**) IDH mutant lower grade gliomas

The ATHENA-generated high-resolution synthetic-sodium MR images exhibited a significant (*P* = 0.0079) improvement in SNR within the T2 hyperintense regions (23.83 ± 9.33) compared to the native sodium MR images (18.20 ± 7.04). Similarly, within the NAWM regions, synthetic-sodium MR images demonstrated a significantly (*P* = 0.0124) higher SNR than the native images (synthetic NAWM-SNR: 14.65 ± 5.68, native NAWM-SNR: 11.10 ± 4.55). Figure 2 (left panel) shows post-contrast T1-weighted, T2-weighted, FLAIR, native sodium, and synthetic sodium images for multiple patients (A–E). The enhancement in image quality was evident in both high- and low-grade gliomas, where the synthetic sodium provided superior structural delineation with reduced noise, enabling better visualization of tumor morphology.

The correlation plots in Fig. 2 (right column) compare synthetic and native sodium MRI signal intensities with data points from contrast-enhancing tumors (green), T2 hyperintense regions (red) and necrosis (blue). The strong linear correlation between native and synthetic sodium concentrations (*P < 0.0001)* suggests that the ATHENA model preserves biologically relevant sodium distribution.

### Validation against native sodium MR imaging

**Fig. 3:**
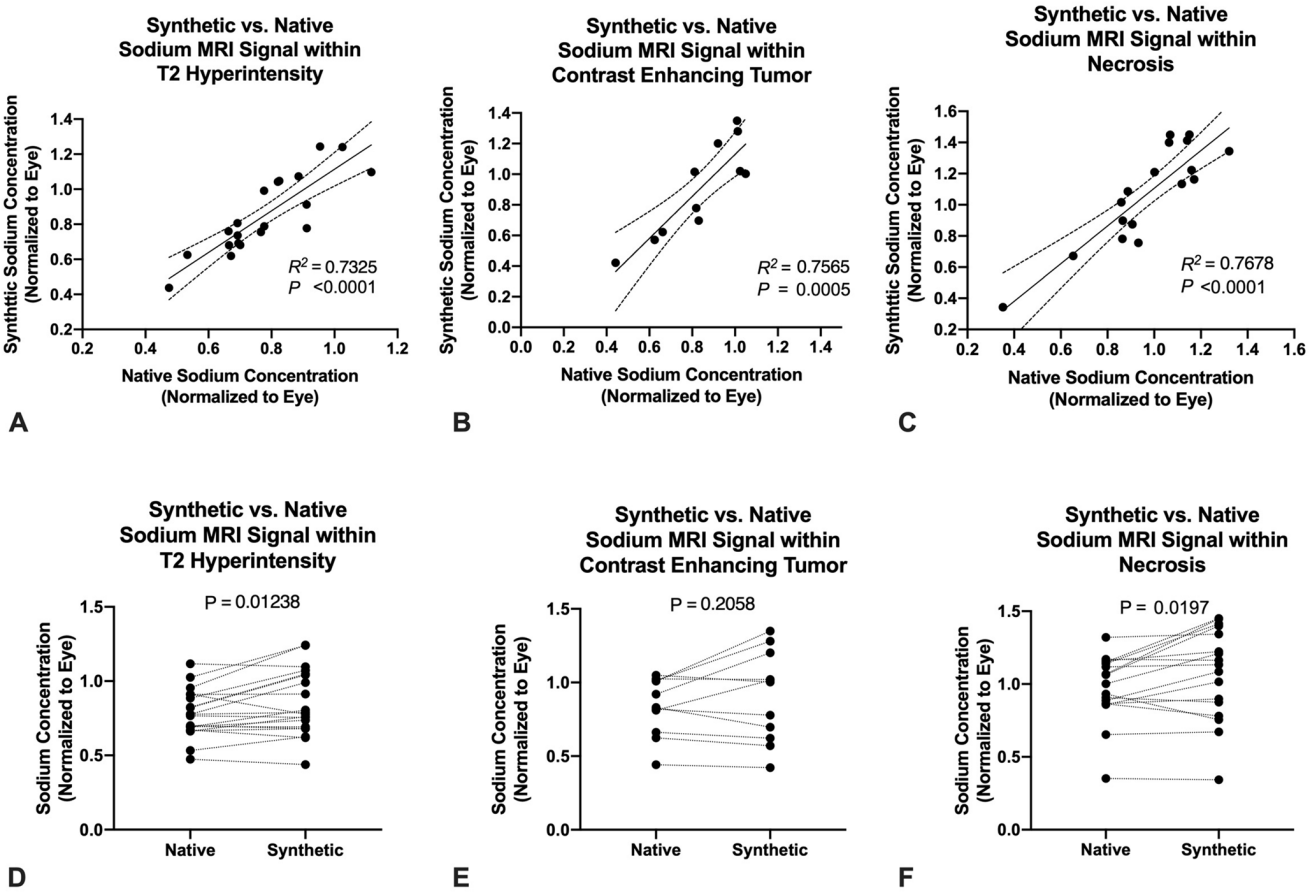
(**A**,** B**,** C**) Pearson’s Linear correlation between synthetic and native sodium MR measurements within (**A**) the T2 hyperintensity, (**B**) contrast-enhancing tumor regions, and (**C**) Necrosis. (**D**,** E**,** F**) Paired t-test comparison of synthetic and native average sodium MRI signal (normalized to the eye) within (**D**) T2 hyperintensity tumor, (**E**) contrast-enhancing tumor, and (**F**) Necrosis Regions

The synthetic-sodium MR signal intensities showed a strong linear correlation with native sodium MR measurements across the glioma cohort within the T2-hyperintense regions (Fig. 3A; *R²=0.7325*,* P < 0.0001*,* slope = 1.188*,* 95% CI=[0.8322*,* 1.543]*), the contrast-enhancing regions (Fig. 3B; *R²=0.7565*,* P = 0.0005*,* slope = 1.371*,* 95% CI=[0.7843*,* 1.957]*) as well as necrosis (Fig. 3C; *R²=0.7678*,* P < 0.001*,* slope = 1.209*,* 95% CI=[0.8433*,*1.557]*), indicating that the GAN model effectively preserved tissue-specific sodium distribution. There is a significant difference between synthetic and native sodium MR values within the T2-hyperintense tumor (Fig. 3D, *P* *= 0.0124*) and necrosis (Fig. 3E, *P* *= 0.0197*) regions, while no significant difference was observed within the contrast-enhancing regions (Fig. 3F, *P* *= 0.2058*), which could be potentially explained by improved delineations of the T2-hyperintense and necrosis VOI and therefore mitigating the partial volume effects in the native low-resolution sodium images. No significant difference was observed between newly diagnosed and recurrent tumors (Supplemental Fig. 3).

### Association with NHE1 expression

**Fig. 4:**
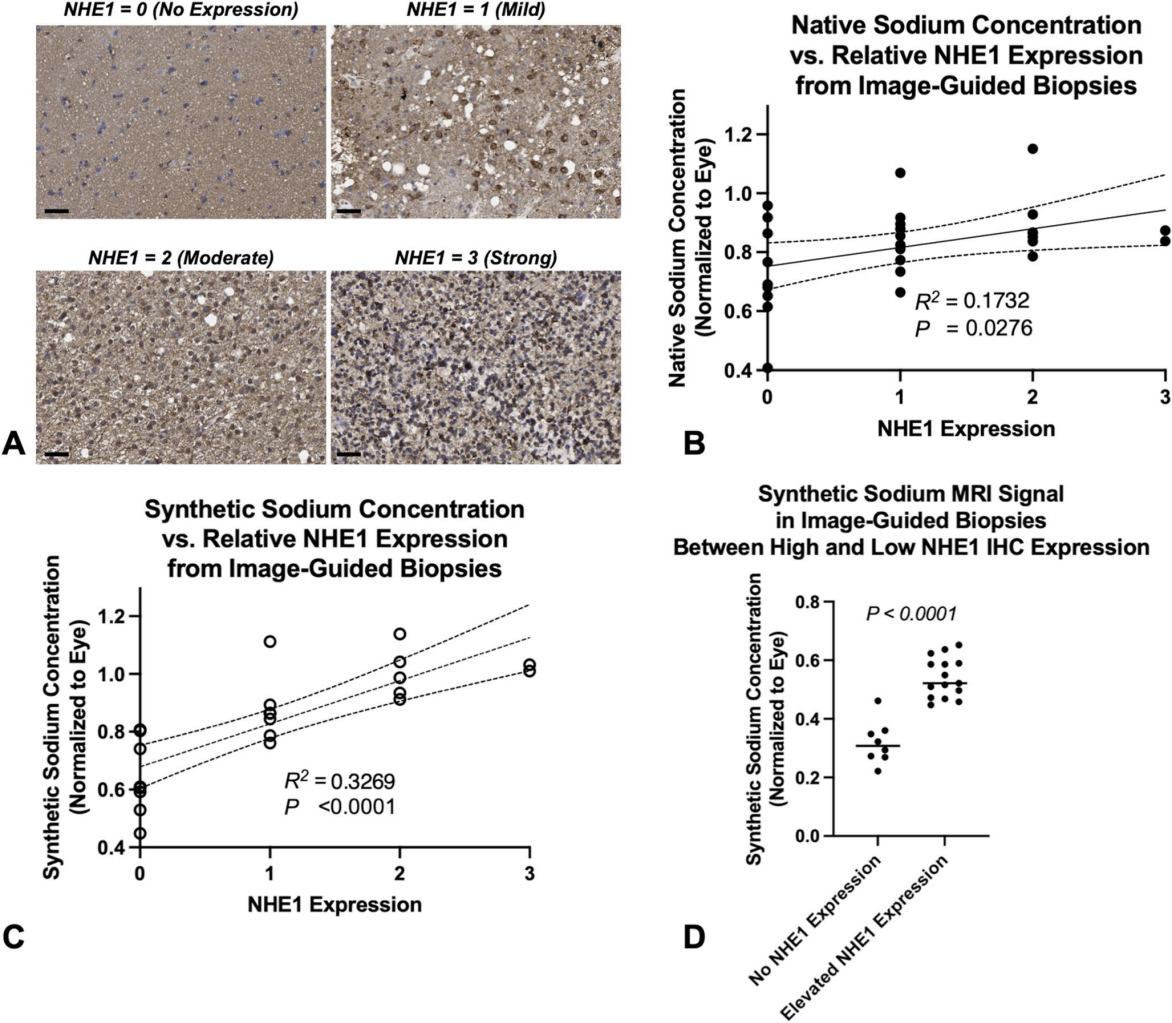
(**A**) Examples of NHE1 immunohistochemistry (IHC) expression levels. (**B**) Spearman’s correlation between native sodium MR signal and NHE1 IHC expression from image-guided biopsies (relative expression). (**C**) Spearman’s correlation between synthetic-sodium MR signal and NHE1 IHC expression from image-guided biopsies (relative expression). (**D**) Comparison of synthetic-sodium MR measurements of image-guided biopsy samples with no NHE1 expression compared with those having notable expression

To further validate the biological relevance of the synthetic-sodium MR images, we analyzed the relationship between sodium signal intensities and relative NHE1 expression levels in tumor tissues (Fig. 4A). Statistical analysis revealed a significant positive correlation between relative NHE1 expression and both the native (Fig. 4B; *R²=0.1731*,* P = 0.0276*) and the synthetic (Fig. 4C; *R²=0.3269*,* P < 0.0001*) sodium MR signal. This was further confirmed by a significant difference in synthetic-sodium MR signal intensity between regions of low and high NHE1 expression (Supplemental Fig. 4; Fig. 4D; *P* < 0.0001).

## Discussion

The current study demonstrates that a physics-informed ATHENA generative adversarial network can be used to successfully reconstruct high-resolution, high-SNR synthetic-sodium MRI images at 3T. Using a modified Pix2Pix architecture with an Attention R2UNet generator, the approach significantly improves image quality while preserving clinically relevant biological information. This advancement creates new opportunities for integrating sodium MRI into clinical practice by overcoming its traditional limitations, which have been extensively documented in previous studies [1, 2, 4–6].

The synthetic-sodium MRI showed significant SNR improvement (native SNR 18.20 ± 7.04; synthetic SNR 23.83 ± 9.33, *P* = 0.0079) (native NAWM-SNR 11.10 ± 4.55, synthetic NAWM-SNR 14.65 ± 5.68, *P* = 0.0124) while maintaining signal fidelity. This enhanced image quality enables better visualization of tumor morphology and improved assessment of tumor microenvironmental properties. Previous attempts to improve sodium MRI have typically relied on hardware solutions such as increasing the magnetic field strength [7, 14], specialized coil designs [18, 19], or ultra-short TE acquisition schemes combining non-cartesian trajectories [15, 16], which are costly and present practical limitations in clinical settings. Nagel et al. [7] demonstrated improved sodium imaging at 7T but acknowledged the limited availability of such systems for routine clinical use. Our computational approach offers a more accessible pathway to high-quality sodium imaging without additional scan time or specialized equipment, aligning with recent trends in applying deep learning to medical imaging enhancement [23].

While the ATHENA model appears to improve image quality and signal-to-noise ratio, systematic discrepancies were observed between the native sodium and synthetic-sodium MR images in certain cases. This difference may reflect a potential bias in the model’s synthetic predictions. Alternatively, the deviations could result from reduced partial volume effects in the synthetic high-resolution sodium images compared to the low-resolution native sodium images, leading to a more accurate representation of tissue sodium concentration. The stronger correlation between synthetic sodium signal and tissue NHE1 expression supports this interpretation. However, in the absence of ground truth high-resolution images from ultra-high field acquisitions, the source of this disparity remains debatable. This bias seems pronounced only in certain patients, possibly indicating variations in tumor pathology or anatomical differences. These findings highlight the need further validation and refinement of the generative model, including validation using ultra-high field sodium images for further refinement of both the generative model and the normalization strategy, potentially incorporating additional physiological priors or calibration techniques, and fine-tuning of the model with additional low-resolution consistency loss, to enhance quantitative accuracy and ensure reliable sodium quantification for clinical applications.

The strong correlation between synthetic and native sodium measurements in both T2-hyperintense (*R²=0.7325*,* P < 0.0001*) and contrast-enhancing regions (*R²=0.7565*,* P = 0.0005*) demonstrates that the ATHENA approach preserves the underlying tissue-specific sodium distribution. Unlike many deep learning approaches that may sacrifice biological accuracy for visual enhancement [22, 34], our physics-informed model maintains the integrity of the sodium signal, crucial for accurate clinical interpretation. This aligns with findings from Zhao et al. [44], who demonstrated that integrating physics constraints into image reconstruction can improve accuracy while preserving tissue-specific information. The anatomical constraints incorporated into our model through edge-detected images ensure that structural details are preserved throughout the reconstruction process, similar to the approach proposed by Haldar et al. [25] for anatomically constrained reconstruction from noisy data.

Perhaps most significantly, the correlation between synthetic-sodium values and NHE1 expression (*R²=0.3269*,* P < 0.0001*) validates the biological relevance of our reconstructed images. The stronger correlation observed with synthetic-sodium compared to native sodium (*R²=0.1731*,* P = 0.0276*) suggests that the improved spatial resolution and SNR may provide a more accurate representation of tumor biology. NHE1 has been established as a critical regulator of intracellular pH and cell volume in gliomas [9, 10], with McLean et al. [11] demonstrating its role in malignant transformation. This biological validation distinguishes our approach from previous computational methods that typically lack verification against molecular markers [24, 26]. The ability to non-invasively assess NHE1 expression—a marker associated with tumor aggressiveness and treatment response as shown by Cong et al. [45]—could provide valuable information for personalized treatment planning without the need for additional invasive procedures.

The clinical implications of our findings extend beyond improved visual quality. While this study establishes the feasibility of generating high-resolution synthetic sodium images to overcome SNR and resolution constraints at 3T, the true clinical impact will come from extending these synthetic maps into quantitative models of total sodium content and compartmental shifts that can directly inform treatment response and patient management. High-resolution synthetic-sodium imaging is a technically enabling step toward future clinical applications that could enhance diagnostic accuracy for brain tumors, particularly in distinguishing tumor types and grades based on their metabolic profiles, as suggested by previous studies on sodium MRI in tumor characterization [8, 12]. Thulborn et al. [46] demonstrated that sodium MRI could differentiate tumor progression from treatment effects, a critical clinical challenge. Additionally, Neto et al. [47] showed that sodium imaging could serve as a biomarker for treatment response assessment, as changes in sodium concentration often precede structural changes visible on conventional MRI. The quantitative nature of our approach allows for objective measurement of these changes over time, potentially providing earlier indications of treatment efficacy than conventional imaging methods [48].

Despite these promising results, our study has several limitations. Firstly, ultra-high field or measured high-resolution sodium images were not available for our cohort due to long acquisition times that made it impractical to acquire them without compromising patient comfort. Additionally, the ATHENA model was trained using single-site data across multiple scanners, which may restrict its generalizability across different vendor platforms and institutional protocols, a common challenge in medical imaging AI [49, 50]. While our dataset of 4,573 proton MR images with synthetic artifacts was substantial, multi-site validation would enhance robustness as demonstrated by Zech et al. [49] in their multi-institutional deep learning study. Our validation cohort of twenty glioma patients, though valuable for initial assessment, represents a relatively small sample size that limits subgroup analyses across different tumor types and grades compared to larger imaging studies [49]. Future studies with larger, multi-institutional cohorts are needed to validate the generalizability and clinical utility of the proposed synthetic sodium imaging approach.

The modified Pix2Pix GAN architecture was crucial to our success, effectively recognizing and correcting complex artifacts while preserving anatomical detail. Similar to work by Armanious et al. [22] and Xie et al. [34], our approach leveraged the strengths of conditional GANs for medical image enhancement. The multi-step training process with progressively increasing artifact complexity contributed to robust artifact removal and image enhancement, a strategy also employed by Oktay et al. [37] in their attention-based models. However, as noted by Cohen et al. [51], the model’s performance could be affected when applied to real-world data not represented in the synthetic training process.

Future work should focus on training ATHENA with multi-institutional datasets to mitigate single-site limitations and validate the model across various scanner settings and patient populations, following best practices outlined by Willemink et al. [52]. Expanding the prospective imaging to a broader cohort, including different tumor grades and subtypes as suggested by Regnery et al. [12], will help better understand how synthetic-sodium imaging can serve distinct clinical needs. Additionally, deep learning frameworks, such as ATHENA, could be integrated with acquisition acceleration techniques to further enhance the efficiency and accessibility of high-resolution sodium imaging. Finally, our physics-informed synthetic image generation approach shows potential for integration with diffusion models [53, 54], which could further enhance analysis of tumor microenvironments and interpretation of sodium imaging data.

## Conclusion

This study evaluates a novel GAN-based framework, ATHENA, to enable high-quality sodium neuroimaging at 3T. By addressing the technical challenges of low SNR and resolution in sodium MRI, our method has the potential to advance the clinical utility of sodium imaging in brain tumor diagnosis and monitoring, with implications for a better understanding of tumor biology.

The ATHENA model generates high-resolution synthetic-sodium MR images that retain critical biological information and demonstrate strong correlations with both native sodium measurements and NHE1 expression. This approach could facilitate better diagnosis, monitoring, and treatment planning for patients with brain tumors. Moreover, the ability to correlate synthetic-sodium images with specific molecular markers opens new avenues for understanding tumor biology and developing personalized treatment strategies, ultimately contributing to improved patient outcomes.

## Electronic supplementary material

Below is the link to the electronic supplementary material.

Supplementary Material 1

## Data Availability

The data that support the findings of this study will be made available upon reasonable request to the corresponding authors. Due to intellectual property considerations, some datasets will be evaluated on a case-by-case basis to ensure appropriate protection of proprietary information while maximizing scientific transparency.
