# Supplementary material for: Super-resolution sodium MRI of human gliomas at 3T using physics-based generative artificial intelligence
Source: J Neurooncol. 2025 Jun 3;174(3):653–65. doi: 10.1007/s11060-025-05094-x (PMC12263758; doi:10.1007/s11060-025-05094-x)
Supplement: Supplementary file 1 — Supplementary Material 1 [file 11060_2025_5094_MOESM1_ESM.docx]

**SUPPLEMENTAL MATERIAL**

**Supplemental Table 1**. MRI acquisition parameters for the training set

|  | | |
| --- | --- | --- |
| **Training data type** | **Number of scans** | **Percentage** |
| Modality |  |  |
| T1-weighted (MPRAGE/SPGR) | 1341 | 29.3% |
| Post-contrast T1-weighted (MPRAGE/SPGR) | 932 | 20.4% |
| T2-weighted (TSE/SPACE) | 1313 | 28.4% |
| T2-FLAIR (TSE/SPACE) | 987 | 21.6% |
| Magnetic Field Strength |  |  |
| 1.5 T | 6 | 0.1% |
| 3 T | 4567 | 99.9% |

|  | **T1-weighted** | | **Post-contrast T1-weighted** | |
| --- | --- | --- | --- | --- |
| **Parameter** | **Mean** | **SD** | **Mean** | **SD** |
| Repetition Time (ms) | 2024.1 | 233.7 | 2022.1 | 267.5 |
| Echo Time (ms) | 2.7 | 0.3 | 2.7 | 0.3 |
| Inversion Time (ms) | 1060.9 | 79.4 | 1065.0 | 76.0 |
| Flip Angle (degree) | 14.72 | 7.50 | 15.17 | 8.50 |
| Slice Thickness (mm) | 1.13 | 0.60 | 1.16 | 0.69 |
| Pixel Spacing (mm) | 0.99 | 0.07 | 0.98 | 0.08 |
| Acquisition type | **Cases** | **Percentage** | **Cases** | **Percentage** |
| 2D | 63 | 6% | 51 | 7% |
| 3D | 1278 | 94% | 881 | 92% |
|  | **T2-weighted** | | **T2/FLAIR** | |
| **Parameter** | **Mean** | **SD** | **Mean** | **SD** |
| Repetition Time (ms) | 4533.3 | 921.5 | 8811.9 | 849.6 |
| Echo Time (ms) | 164.5 | 120.0 | 95.4 | 36.6 |
| Inversion Time (ms) | ~~-~~ | ~~-~~ | 2475.4 | 95.3 |
| Flip Angle (degrees) | ~~-~~ | ~~-~~ | 146.86 | 15.46 |
| Slice Thickness (mm) | 2.49 | 0.95 | 2.81 | 0.76 |
| Pixel Spacing (mm) | 0.67 | 0.23 | 0.73 | 0.21 |
| Acquisition type | **Cases** | **Percentage** | **Cases** | **Percentage** |
| 2D | 991 | 73% | 865 | 88% |
| 3D | 306 | 37% | 122 | 12% |

- **Note**: Some key parameters in the DICOM headers were missing in 32 scans (< 1%).

**
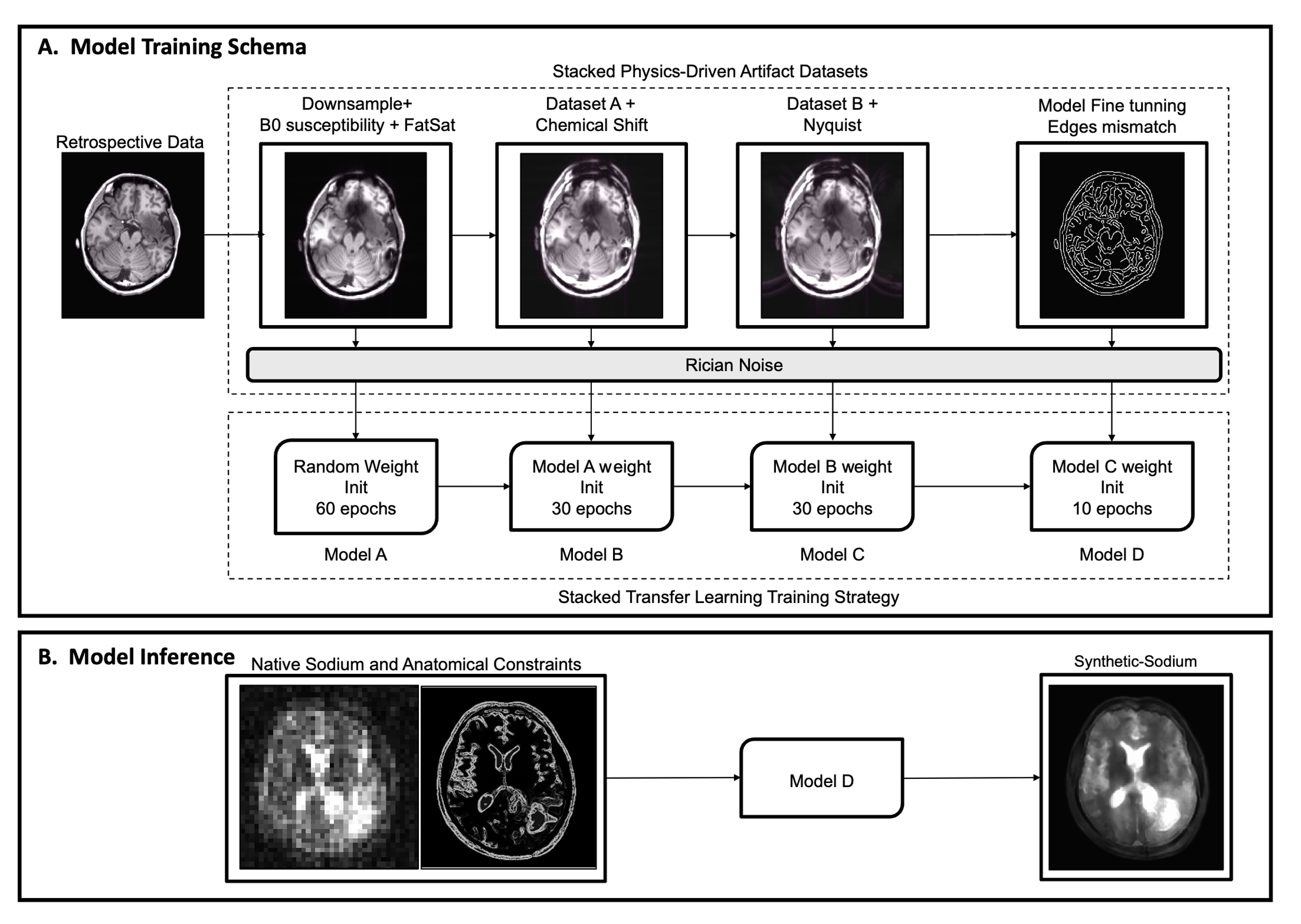
**

**Supplemental Figure 1. (A)** Sequence diagram for the training portion of the “stacked transfer learning” approach, with an example case of simulated artifacts sequentially added on. The model was trained with a stacked approach, starting with simple artifacts and progressively increasing the complexity of the artifacts. The trained weights from the previous model were used to initialize the next model. **(B)** Sequence diagram for the testing of the “stacked transfer learning” final model on sodium MRI.


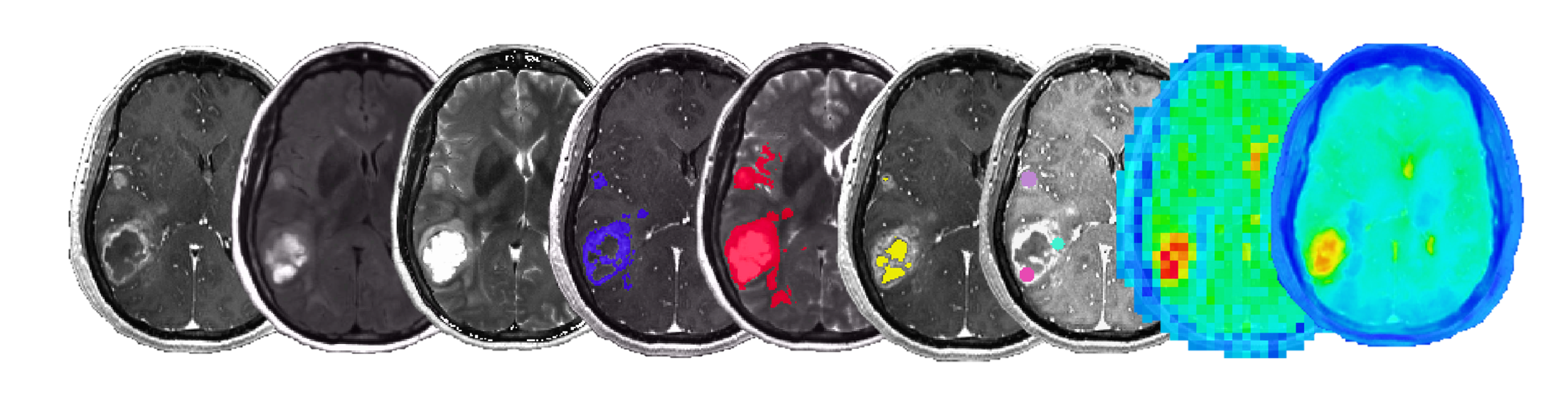


**Supplemental Fig. 2.**  Example case. [Left to Right] Pre-contrast T1-weighted, post-contrast T1-weighted; FLAIR; T2-weighted; contrast-enhancing tumor VOI; T2-hyperintensity VOI; Necrosis VOI; Image-guided biopsies VOI, native sodium, synthetic-sodium.

**Supplemental Fig. 3.**  Comparison of sodium contrast within tissue compartments between newly diagnosed and recurrent patients.


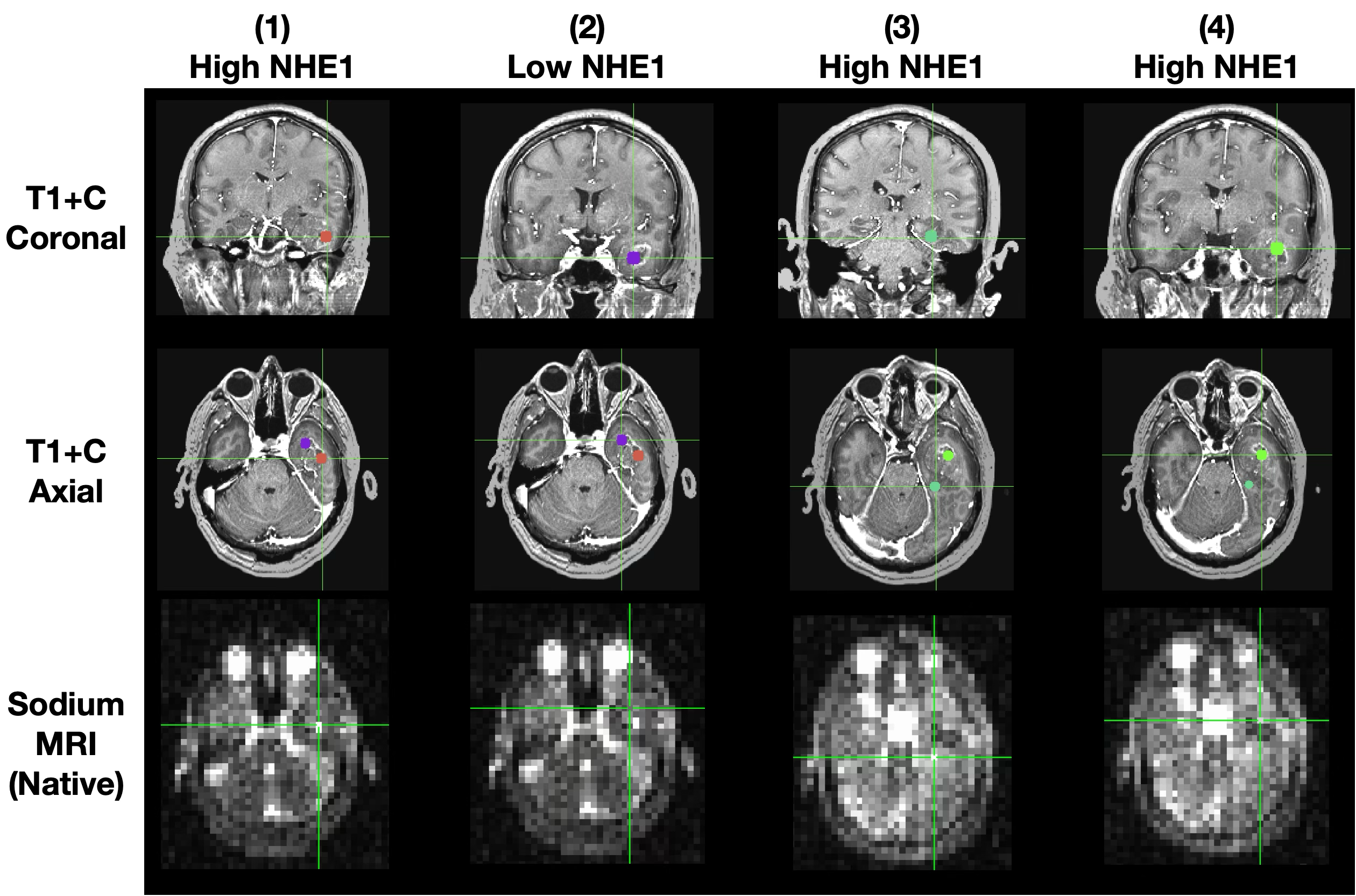


**Supplemental Fig. 4.** Example biopsy sites from Patient 1 showing areas of high and low NHE1 expression within the same enhancing lesion.
